# Supplementary figures and images for: Multimodal CustOmics: A unified and interpretable multi-task deep learning framework for multimodal integrative data analysis in oncology
Source: PLoS Comput Biol. 2025 Jun 17;21(6):e1013012. doi: 10.1371/journal.pcbi.1013012 (PMC12173418; doi:10.1371/journal.pcbi.1013012)

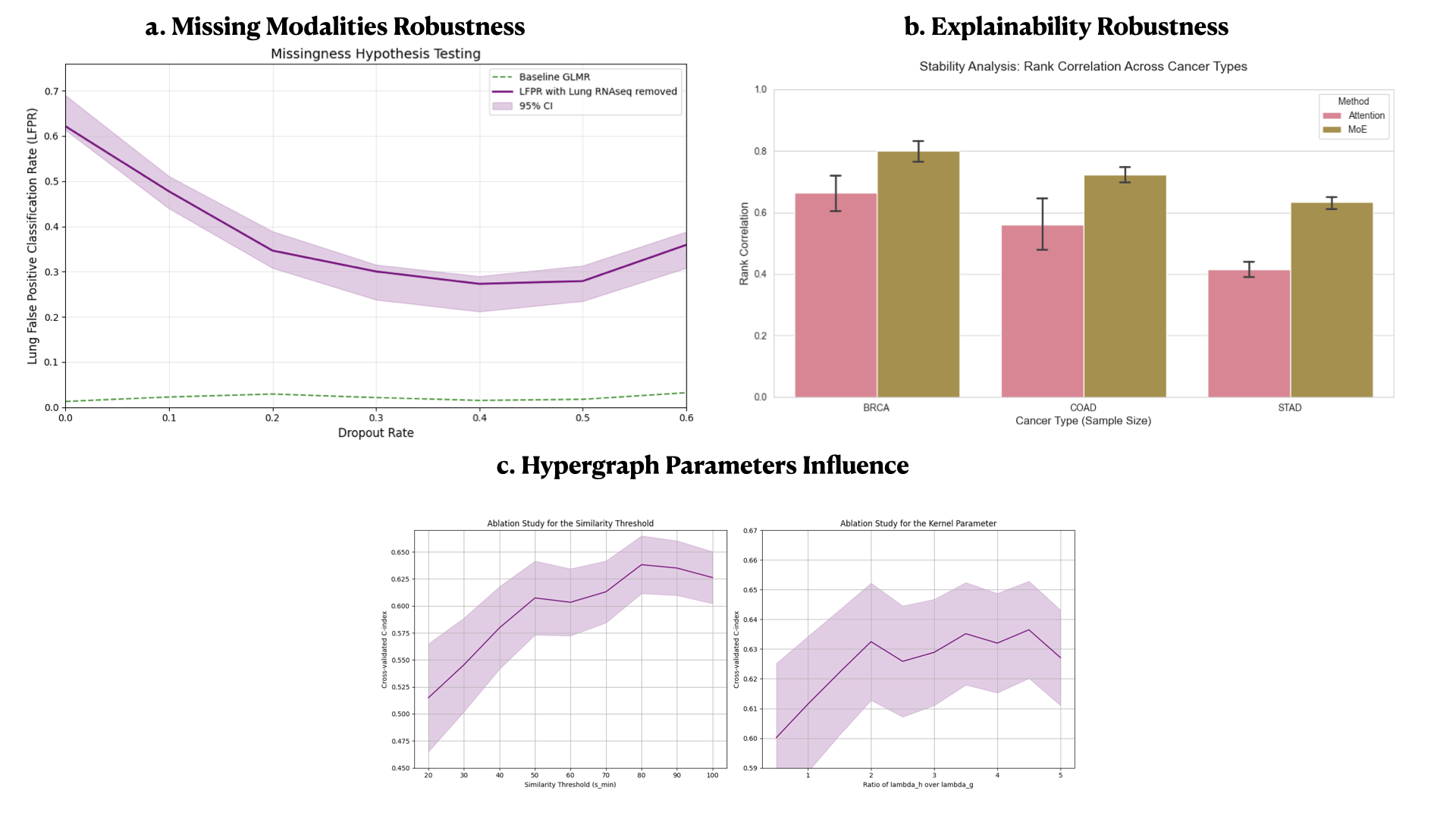

Supplement: S1 Fig — a. Evolution of Lung False Positive Rate (LFPR) across different RNAseq dropout rates during testing, with baseline Missing at Random (MAR) performance (green dashed line) and 95% confidence intervals (shaded area). b. Rank correlation comparison between MoE and attention-based feature importance across three TCGA cancer types (BRCA, COAD, STAD) with error bars indicating standard deviation. c. Ablation studies showing model performance sensitivity to hypergraph construction parameters: similarity threshold (left) and kernel parameter ratio (right), with 95% confidence intervals (shaded areas). (TIF) [file pcbi.1013012.s008.tif]
