# Supplementary material for: Multimodal CustOmics: A unified and interpretable multi-task deep learning framework for multimodal integrative data analysis in oncology
Source: PLoS Comput Biol. 2025 Jun 17;21(6):e1013012. doi: 10.1371/journal.pcbi.1013012 (PMC12173418; doi:10.1371/journal.pcbi.1013012)
Supplement: S1 Text — Ideas and methodology behind SHAP values. (PDF) [file pcbi.1013012.s009.pdf]

Text S1: SHAP Values

April 7, 2025

SHAP (Shapley Additive exPlanations) values provide a method to explain the output of any machine learning model grounded in cooperative game theory. The concept is based on Shapley values, which distribute 'payouts' fairly to 'players' in a coalition. In machine learning, 'payouts' are the model's predictions, and 'players' are the input features.

The Shapley value, a core concept in cooperative game theory, offers a fair distribution of payoffs among players based on their contribution. For a player i in a set N of players, the Shapley value is mathematically defined as:

$$\phi_i(v) = \sum_{S \subseteq N \setminus \{i\}} \frac{|S|!(|N| - |S| - 1)!}{|N|!} \left(v(S \cup \{i\}) - v(S)\right) \tag{1}$$

where S is a subset of players excluding i, and $v(S)$ is the value function of the coalition S.

In machine learning, SHAP values interpret the contribution of each feature to predicting a particular instance. The SHAP value of feature j for a prediction instance x in a feature set X is calculated as:

$$\text{SHAP}_j(x) = \sum_{S \subseteq X \setminus \{j\}} \frac{|S|!(|X| - |S| - 1)!}{|X|!} \left(f_x(S \cup \{j\}) - f_x(S)\right) \tag{2}$$

where $f_x(S)$ represents the output of model f when the feature set S is present for instance x.

SHAP values offer several advantages in model interpretation:

- **Consistency:** If the model changes such that a feature's contribution increases or remains the same, its SHAP value should not decrease.
- **Local Accuracy:** The sum of SHAP values for all features plus a base value equals the model prediction.
- **Global Interpretability:** Aggregating SHAP values across a dataset provides an overview of the model's behavior.

Applicable to any machine learning model, SHAP values are precious in areas requiring high transparency, such as healthcare, finance, and criminal justice. They enhance the understanding and trustworthiness of AI-driven decisions by offering detailed insights into the contributions of individual features.
