# Supplementary material for: Multimodal CustOmics: A unified and interpretable multi-task deep learning framework for multimodal integrative data analysis in oncology
Source: PLoS Comput Biol. 2025 Jun 17;21(6):e1013012. doi: 10.1371/journal.pcbi.1013012 (PMC12173418; doi:10.1371/journal.pcbi.1013012)
Supplement: S2 Text — Fundamentals of Survival Outcome Prediction Tasks. (PDF) [file pcbi.1013012.s010.pdf]

Text S2: Survival Analysis

April 7, 2025

Survival analysis is a branch of statistics focused on analyzing time until an event of interest, commonly used in diverse fields like medicine, biology, and engineering. This analysis deals with the challenge of censored data, where the event time for some subjects is unknown.

At the core of survival analysis is the **Survival Function** $S(t)$, representing the probability of survival beyond time t. Defined as $S(t) = P(T > t)$, where T represents the survival time, this function is characterized by its non-increasing nature, starting from $S(0) = 1$ and approaching zero as time goes to infinity.

Complementing the survival function is the **Hazard Function** $\lambda(t)$, which provides the instantaneous event rate at time t, conditional on survival until that point. It is mathematically expressed as:

$$\lambda(t) = \lim_{\Delta t \to 0} \frac{P(t \leq T < t + \Delta t | T \geq t)}{\Delta t} \tag{1}$$

This function plays a pivotal role in understanding the dynamics of the time-to-event process.

Handling incomplete data due to censoring is a crucial aspect of survival analysis. Censoring occurs when the information about an individual's event time is incomplete, and it comes in various forms, such as right-censoring, left-censoring, and interval-censoring.

The **Kaplan-Meier Estimator** is a cornerstone in non-parametric survival analysis, used for estimating the survival function from life-table data. Given observed survival times, this estimator calculates the survival probability at different time points, accounting for censored data.

For examining the relationship between survival time and one or more predictor variables, the **Cox Proportional Hazards Model** is extensively used. It is a semi-parametric model that defines the hazard function as:

$$\lambda(t|X) = \lambda_0(t) e^{\beta' X} \tag{2}$$

where $\lambda_0(t)$ is the baseline hazard, X represents covariates, and β is the coefficient vector.

In cases where a specific distribution of survival times is assumed, **Parametric Models** like exponential, Weibull or log-normal models are employed. These models offer greater flexibility but require conforming to certain distributional assumptions.

Survival analysis is indispensable across various applications, from assessing patient survival in medical studies to analyzing failure times in engineering. Its capacity to handle censored data and model time-to-event relationships makes it a vital statistical analysis and research tool.

In summary, survival analysis provides a comprehensive framework for analyzing and predicting the time until an event of interest occurs. Using different statistical methods, it addresses the complexities of censored data and offers insights into the factors influencing survival times.
