# Supplementary material for: Multimodal CustOmics: A unified and interpretable multi-task deep learning framework for multimodal integrative data analysis in oncology
Source: PLoS Comput Biol. 2025 Jun 17;21(6):e1013012. doi: 10.1371/journal.pcbi.1013012 (PMC12173418; doi:10.1371/journal.pcbi.1013012)
Supplement: S3 Text — Methodology behind the standard mixture-of-expert (MoE) framework. (PDF) [file pcbi.1013012.s011.pdf]

Text S3: Mixture of Experts

April 7, 2025

The Mixture of Experts (MoE) model is a versatile approach in statistical modeling and machine learning, designed to capture complex patterns in data by combining the predictions of multiple 'expert' models. This approach is particularly effective in scenarios where different subsets of data are best explained by different types of models.

Central to the MoE model is the idea of partitioning the input space into regions, each dominated by an expert, which is typically a separate model or learner. The combination of these experts' predictions provides a comprehensive output that reflects the nuances of the entire dataset.

The architecture of a MoE model comprises two main components:

- **Experts**: Each expert is a model (like a regression or a neural network) trained on the dataset. In a MoE model, multiple experts are trained to specialize in different parts of the input space.
- **Gating Network**: This is a probabilistic model that determines the weight or influence of each expert for a given input. The gating network's output is a set of weights that sum to one, indicating the proportion of contribution from each expert.

Mathematically, the output of a MoE model for an input x is a weighted sum of the outputs from the experts. If $y_i(x)$ is the output of the i-th expert and $g_i(x)$ is the weight assigned by the gating network for this expert, the overall output $Y(x)$ is given by:

$$Y(x) = \sum_i g_i(x) y_i(x) \tag{1}$$

where $\sum_i g_i(x) = 1$, and $g_i(x)$ is typically determined using a softmax function over the gating network outputs.

One of the key advantages of the MoE model is its flexibility. By combining experts that are proficient in different regions of the input space, the MoE model can approximate a wide variety of functions, making it suitable for complex tasks like pattern recognition, time-series forecasting, and nonlinear regression.

In practical applications, the training of a MoE model involves optimizing both the parameters of the experts and the gating network, typically using gradient-based methods. This process involves balancing the specialization of each expert with the overall coherence of the model.
