# Supplementary material for: Multimodal CustOmics: A unified and interpretable multi-task deep learning framework for multimodal integrative data analysis in oncology
Source: PLoS Comput Biol. 2025 Jun 17;21(6):e1013012. doi: 10.1371/journal.pcbi.1013012 (PMC12173418; doi:10.1371/journal.pcbi.1013012)
Supplement: Attachment — Submitted filename: Customics___Reviews-2.pdf [file pcbi.1013012.s012.pdf]

Revision (2nd)
Response to reviewers
for manuscript PCOMPBIOL-D-24-01276

Multimodal CustOmics: A Unified and Interpretable Multi-Task Deep Learning Framework for Multimodal Integrative Data Analysis in Oncology

From the reviewers' and the editor's comments, the main point to improve in the manuscript was its clarity. We made multiple corrections to the text and added more details on some notions to obtain a more self-contained paper.

Contents

1 General Comments **1**

2 Reviewer 1 **3**

3 Reviewer 2 **13**

1 General Comments

General Comments: We thank all reviewers for their valuable insights that helped improve our manuscript's clarity and technical depth. Regarding the key points highlighted in the reviews, we have provided detailed clarifications about our model's handling of missing modalities, explaining how dropout is implemented. We have tested the model's robustness to Missing Not At Random (MNAR) scenarios through a systematic experiment where RNAseq data was purposefully removed from Lung Cancer samples during training while keeping other modalities intact. Our analysis of the Global Lung Misclassification Rate (GLMR) across varying dropout rates revealed that moderate levels of missingness during training (around 40%) actually help the model rely more on biological signals from remaining modalities, though there remains some level of bias that cannot be completely overcome. We have also enhanced the explanation of our hypergraph structure, confirming that nodes represent grouped patches connected by hyperedges based on morphological similarity thresholds, while simplifying notation for better readability. On the explainability front, we have demonstrated how our MoE-based approach provides robust interpretability by modeling biological hier-

archies through specialized expert networks. This was empirically validated through stability analysis across multiple cancer types, showing consistently higher rank correlations between weights from different training folds compared to traditional attention mechanisms. These clarifications strengthen the technical foundation of our work while making it more accessible to readers.

In the following, we answer each specific question and comment of the reviewers in detail.

2 Reviewer 1

Reviewer's comment: The motivation of using a hypergraph autoencoder is unclear. Why is this necessary? The ablation study in Table S4 shows that alternative encoders (e.g. based on a regular graph embedding, A3, or a hierarchical mixture of experts, A4) perform similar, if not better, than the hypergraph approach. Overall, the rationale behind this modelling decision could be improved, e.g. why is a hypergraph necessary? why is the hypergraph constructed based on morphological similarity versus spatial proximity?

Our reply:
Our selection of the hypergraph autoencoder stems from its ability to capture higher-order relationships that traditional graph-based approaches cannot detect. By encoding relationships between multiple entities simultaneously, hypergraphs enable both robust local and region-wise embeddings.
Regarding similarity measures, we opted for morphological similarity over spatial proximity as it better reflects the underlying latent structure of the data. This approach can identify meaningful relationships between entities that share important traits but may be spatially separated.
Our ablation study, presented in Table S4, reveals that while the hypergraph approach may underperform compared to transformer architectures in certain scenarios, it demonstrates better performance in integrating Whole Slide Imaging (WSI) with multi-omics data. This advantage is particularly relevant given the complex requirements of multimodal integration.
The hypergraph framework offers advantages through its reduced parameter count compared to transformer-based models, resulting in lower complexity. This aligns with Multimodal CustOmics' vision of dividing multimodal representation learning into smaller, interpretable strata. In multimodal setups, our lighter approach achieves comparable or improved results compared to transformers, supporting its implementation in our model.
To provide greater clarity on these points, we have expanded Section 2.1.1 to include our motivation for using hypergraphs and added a detailed discussion in the manuscript's discussion section. Furthermore, we have included a comparison of parameter counts between hypergraph and transformer approaches in the supplementary materials.

◊

Reviewer's comment: It is unclear how the model handles different modalities (i.e. under what missingness assumption it operates) and whether it is affected by artifacts. Can you demonstrate that the missingness pattern is not predictive of the downstream task? Is it possible to prove that, if certain conditions are fulfilled, the method will work as intended? (e.g. if the data is missing not at random at train time, can you guarantee that the method will generalize well at test time?). Some studies have found

that JPEG quality can have a strong confounding effect on histopathological feature representation and tissue classification in the TCGA cohort (see Fu et al., "Pan-cancer computational histopathology reveals mutations, tumor composition and prognosis"). To what extent do these confounding factors influence the method's predictions?

Our reply:
It is an interesting comment. Our model operates under a Missing at Random (MAR) assumption but is flexible enough to adapt to Missing Not at Random (MNAR) settings with proper hyperparameter tuning. To evaluate this, we designed an experiment where RNAseq data was systematically removed from Lung Cancer samples during training while keeping CNV and methylation data intact. During testing, we randomly removed RNAseq data from other tumor types at varying dropout rates (0% to 60%) and measured the Lung False Positive Classification Rate (LFPR), which quantifies the proportion of non-Lung samples incorrectly classified as Lung Cancer. We computed confidence intervals using bootstrap resampling over 1000 iterations to assess the uncertainty of the observed misclassification rates. Our results show that the model indeed learns to use missingness as a shortcut, with high LFPR at low dropout rates. The misclassification rate reaches its minimum around 40% dropout, suggesting that moderate levels of missingness during training force the model to rely more on biological signals from remaining modalities. However, even at this optimal point, the GLMR remains significantly higher than the baseline performance (when RNAseq is missing at random during training), indicating that the model cannot completely overcome this learned bias.

◊

Reviewer's comment: The benchmark could be improved to account for state-of-the-art methods that have recently been released. For example, a recently introduced state-of-the-art method (HEALNet) addresses the same multi-modality integration goal. Could the authors include this method in their benchmark? (see Hemke et al., "[HEALNet: Hybrid Multi-Modal Fusion for Heterogeneous Biomedical Data"). This and potentially other methods should be included in the benchmark to claim state-of-the-art performance.](https://arxiv.org/abs/2311.09115)

Our reply: Thank you for suggesting including this recent method in our benchmark. In response, we have integrated HEALNet (Hemke et al., NeurIPS 2024) into the benchmark in the revision. While this model was only recently accepted to NeurIPS we recognize its relevance and have updated our analysis accordingly.
By including HEALNet, we observed slight but consistent improvements in performance by Multimodal CustOmics across key metrics. These results reaffirm the robustness of our approach while situating it within a competitive landscape of state-of-the-art methods. The updated results and discussions have been incorporated into the revised manuscript and supplementary materials.

Your suggestion allowed us to strengthen the benchmarking and further validate our claims.

◊

Reviewer's comment: The motivation and advantages of the proposed interpretability method are unclear. What is the advantage of this approach over existing interpretability methods that already account for the architecture of the predictive model? Can the authors show the advantages of the proposed interpretability approach over existing methods? For example, what is the advantage of weighting SHAP values by pathway enrichment scores (e.g. with respect to using plain SHAP values)?

Our reply: We believe that our method provides several advantages over existing approaches. While traditional methods like attention mechanisms can provide feature importance, our MoE-based approach explicitly models the hierarchical nature of biological systems by learning specialized expert networks for different pathways and spatial regions. This specialization is particularly important when working with limited samples, where we need stable and reliable interpretations of the model's decisions. To test this hypothesis, we conducted a systematic stability analysis across three TCGA cancer types with varying sample sizes (BRCA n=1098, COAD n=456, and STAD n=375) using a fixed test set and K-fold cross-validation to compare the consistency of feature importance across different training splits. This analysis revealed that our MoE-based approach produces more stable pathway importance scores compared to attention mechanisms, particularly in datasets with limited sample sizes like STAD. The rank correlation between weights from different training folds was consistently higher for MoE ($\rho \sim 0.75 - 0.82$) compared to attention ($\rho \sim 0.45 - 0.65$), with the gap widening as sample size decreased. From a practical perspective, this stability appears crucial for biological interpretation, as it suggests that the pathways identified as important by our method are more robust to variations in the training data, which is particularly valuable in cancer genomics where sample sizes are often limited and the reliability of feature importance is critical for downstream analysis and clinical interpretation.

◊

Reviewer's comment: L70: "The method outputs the importance score of each gene for each molecular source, both independently and in association with other modalities". To what extent can we trust these scores? Do you have any experiments showing that the method indeed recovers known biomarkers? What does "in association with other modalities" mean exactly?

Our reply: It is indeed a critical aspect to assess. Multiple experiments have shown that the method recovered known biomarkers for different tasks, including FBN2's role in suppressing metastasis (*Mahdizadehi et al.*), established inflammatory signature markers (*Zhao et al.*), and validated spatial interactions between estrogen response and TILs (*Loi et al.*). These findings were extensively listed and detailed in the results section.

◊

Reviewer's comment: L74-76: "This score has also been extended to account for spatial correlations in the histopathology slide and reflects the importance of the interaction between spatial regions of the WSI and pathways". How has the score been extended to account for spatial correlations?

Our reply: This extension actually refers to the multimodal Interaction Score developped in section 2.2.2. The extension has been made possible thanks to the Hierarchical Mixture of Experts that can output importance weights for each region along each pathway.

◊

Reviewer's comment: Fig 1: How is the pathway aggregation carried out? Does this method require prior pathway information or is it purely data-driven?

Our reply: This method takes prior pathway information as input. This is important to be able to drive the model with biologically relevant information depending on the case study. We tried to clarify this point.

◊

Reviewer's comment: Fig 1, caption: "Phase 1 learns the weights of each region inside a pathway" -¿ did the authors intend to say "Phase 1 learns the weights of each pathway inside a region"?

Our reply: Our model represents the data as a tensor, where one dimension corresponds to pathways and the other to tissue regions within the Whole Slide Image (WSI). The hierarchical mixture of experts (MoE) operates in two phases to progressively build a biologically meaningful and interpretable representation.
In Phase 1, the MoE operates along the regions axis. This allows the model to learn weights for tissue regions, aggregating their contributions to form a representation for each pathway. By focusing on the interaction between regions and each pathway,

this phase ensures that the resulting pathway representation is informed by the most relevant tissue regions, for each pathway.
In Phase 2, the MoE operates along the pathways axis. The pathway representations constructed in Phase 1 are further aggregated to build a global representation of the WSI. This final step integrates information across all pathways, weighted by their importance, to provide a comprehensive and biologically meaningful summary of the WSI.
By structuring the data as a tensor and sequentially applying MoE along its dimensions, the model effectively captures localized interactions at the tissue-region level and global interactions at the pathway level. We have updated the manuscript to provide a clearer explanation of this methodology by using terms more precise than "region inside a pathway".

◊

Reviewer's comment: L96-97: How is the ResNet-18 trained via contrastive learning? Details seem to be missing.

Our reply: The ResNet-18 was pretrained in the paper from Ciga et al., it is based on contrastive learning trained on multiple histopathology patch slides from multiple datasets. In this study, we used their pre-trained model as a feature extractor without training it ourselves. More details can be found in their original paper.

◊

Reviewer's comment: L106-L114: How are the patches with high similarities grouped into clusters. Did the authors used a threshold based on the path similarity provided by the kernel? If so, how is this threshold selected? How are λ_h and λ_p defined?

Our reply: Thank you for your question regarding our adaptive clustering approach. The patches with high similarities are grouped into clusters using a threshold defined based on the similarity provided by the kernel. Specifically, this threshold has been extensively studied and validated for the same datasets in the Hyper-adaC paper, which provides a robust foundation for our approach. Therefore, we adopted the same hyperparameters for the similarity threshold as defined in Hyper-adaC. To make things clearer, we added the study for hyperparameter search in the supplementary materials.

◊

Reviewer's comment: L114-L117: How is $\mathcal{P}_i$ defined? Is the clustering algorithm applied to further group the patch groups from the previous step into K categories? Is the interpretation by the pathologist a necessary step to select relevant regions, or is this rather a byproduct of the methodology? (i.e. the identified regions are amenable to interpretation by histopathologists)

Our reply:
$\mathcal{P}_i$ represents the new set of patches obtained through the adaptive clustering process, where high-similarity patches are grouped based on a predefined threshold. After this step, we apply a K-Means algorithm to further cluster these patch groups into K categories, representing distinct regions of interest within the Whole Slide Image (WSI).
The interpretation by pathologists is not necessary for the model to function; it is included in the paper to enhance the interpretability of the identified regions. The methodology itself operates independently of any manual intervention, but the resulting clusters align with biological and histopathological relevance, making them easily interpretable by domain experts. This integration of interpretability ensures that the model's outputs are both actionable and aligned with real-world applications.
We have clarified this in the revised manuscript in Section 2.1 to better communicate this distinction.

◊

Reviewer's comment: L118-L121: Am I right in thinking that nodes in the hypergraph are "grouped patches" and that hyperedges group these patches whenever their morphological similarity exceeds a threshold? I recommend 1) clarifying these points in the text, 2) removing unnecessary symbols (e.g. are G_i, V_i, and E_i used anywhere else in the text?), and 3) including a clear definition of each symbol (e.g. p_j and $\mathcal{P}_i$).

Our reply: Your understanding is correct - the nodes in the hypergraph represent grouped patches, and hyperedges connect patches that exceed a morphological similarity threshold. We appreciate the suggestions and have clarified these points in the text, remove the unused notation (G_i, V_i, and E_i), and add clear definitions for all symbols including p_j and $\mathcal{P}_i$. These changes will improve the readability and technical clarity of the manuscript.

◊

Reviewer's comment: L129-131: How are the P gene sets determined? How are the variational autoencoder models trained?

Our reply: Thank you for these important clarification questions. The P gene sets are provided as input to the model and can represent different biological functionalities depending on the clinical question being investigated - including general signatures, hallmarks, or immunity signatures. Regarding the training of variational autoencoders, we use a mixed-integration strategy that bridges the gap between joint and late integration approaches, as extensively detailed in Benkirane et al. We clarified these points in the revised manuscript.

◊

Reviewer's comment: L131-133: "The encoding networks are designed to accept inputs from all omic sources about each gene set". How are these inputs represented? Could the authors clarify how the different omics modalities need to be represented? e.g. how did they align the CNV and methylation data with RNA-seq?

Our reply: The inputs from different omic sources are represented as follows: RNA-seq data as normalized expression values, CNV data as segment mean values, and methylation as beta values. To align these modalities, we map all features to gene-level representations using canonical annotations - specifically, CNVs are mapped to genes based on their genomic coordinates using the UCSC genome browser annotations, while methylation probes are mapped to their corresponding genes using the manufacturer's annotations (typically within the promoter or gene body regions). We added these technical details to the manuscript to ensure reproducibility.

◊

Reviewer's comment: L140-L141: Can the "bilinear fusion operator" be described in more detail? What are the advantages with respect to other standard modelling choices such as cross-attention?

Our reply: The "bilinear fusion operator" used in our work is inspired by the low-rank bilinear pooling method described in Kim et al. ("Hadamard Product for Low-rank Bilinear Pooling", https://arxiv.org/pdf/1708.01471). This operator is designed to combine information from two modalities by capturing interactions between their respective features in a structured and efficient manner.
In our model, this operator allows for the integration of multimodal data by focusing on the relationships between features from different modalities. It is computationally efficient and well-suited for high-dimensional data, ensuring that interactions are preserved without introducing excessive complexity.
Compared to methods like cross-attention, the bilinear fusion operator offers several advantages. It avoids the high computational and memory demands of cross-attention mechanisms, particularly when dealing with large datasets or high-dimensional fea-

tures. Furthermore, it provides a direct and structured way of combining modalities, making it a simpler yet effective alternative for tasks where capturing pairwise interactions is sufficient.
We have clarified the purpose and advantages of the bilinear fusion operator in Section 2.1.3 of the revised manuscript.

◊

Reviewer's comment: L150: What is the meaning of the sentence after "where (...)". Did the authors want to say that the weights must sum to 1?

Our reply: Thank you for highlighting this, indeed there was a mistake in this sentence. It has been modified in the revised version for more clarity by: "where w_k^p represents the learned importance weights for each spatial region associated with the pathway, and $\sum_{k=1}^{K} w_k^p = 1$ ensures that these weights form a valid probability distribution".

◊

Reviewer's comment: L155: What is the meaning of $(w_p)p$? There seems to be some notation issues.

Our reply: In line with the previous question, we have addressed this inconsistency in the revised version of the manuscript by including the missing explanation to clarify the meaning of this notation.

◊

Reviewer's comment: L171-L177: How are the modalities dropped out, exactly? In Section 2.1.2, it is mentioned that a VAE is used to encode pathway-level features using inputs from all omic sources. In what part of the architecture is the modality dropout performed?

Our reply: Thank you for suggesting this clarification about the dropout implementation. For the omic modalities, the dropout is performed within the variational autoencoders at the input layer, randomly masking entire omic modalities during training. For the WSI modality, the dropout is implemented differently - it occurs before the multimodal fusion network. These elements have been clarified in the paper.

◊

Reviewer's comment: L238-L242: Why is the multiphase training strategy required? Can the authors provide an ablation study showing the benefits of such approach?

Our reply: The multiphase training approach is essential for achieving stable and meaningful model convergence. When attempting to optimize all components simultaneously, we observed significant instability in the learned weights, as the model struggles to balance the different learning objectives across modalities and hierarchical levels. By breaking down the training into phases, we allow each component to establish robust foundations before integrating them. This is particularly crucial for our hierarchical mixture of experts, where the pathway-level representations need to be well-formed before attempting to learn cross-modal interactions. Without this staged approach, we found that the model often converges to suboptimal solutions where the interpretability of the weights is compromised, making it harder to extract meaningful biological insights from the learned representations. As for the ablation study, this one has been already done through Table S4c.

◊

Reviewer's comment: L257: A recently introduced state-of-the-art method (HEALNet) addresses the same multi-modality integration goal. Could the authors include this method in their benchmark? [HEALNet: Hybrid Multi-Modal Fusion for Heterogeneous Biomedical Data](https://arxiv.org/abs/2311.09115)

Our reply: Thank you for pointing out this paper, we added the method in the revised version of the manuscript.

◊

Reviewer's comment: L332: What is the meaning of "gene segmentation"?

Our reply: In this sentence, by "gene segmentation" we meant the affiliation of genes to corresponding pathways. But you are right, the word "segmentation" may be misleading so we changed it in the revised version of the manuscript by "which set of pathways is chosen".

◊

Reviewer's comment: Figure 2: What do the different colors in the panel before 2c represent?. Figure 2c: How was the spatial enrichment analysis carry out, and what

are the observations/conclusions for that panel? Figure 2e: how was the differential analysis carried out? My understanding is that these cell distributions were calculated based on the attention maps of Figure 2c, is this correct? How do these cell fractions align with our understanding of breast cancer subtyping?

Our reply: Figure 2: For this panel showing the slide of the patient to consider, the colored contour is only here to make sure that tissue segmentation has been correctly carried out for the analysis and that the extracted spatial tissues considered for the analysis are only taking into account the tissue and as little information from the background as possible. Figure 2c: In this panel, we show a heatmap of the spatial regions for which the interaction with specific pathways are important for the classification/survival. This is indeed not an ”enrichment analysis” per say but rather an importance heatmap extracted from the results of the hierarchical mixture of experts. Figure 2e: Here, using automatic segmentation and classification state-of-the-art tools, we extract cell types for each cell in both high and low attention regions (defining a threshold of 0.7 for differentiating the populations) and we perform a standard t-test comparing the cell population for each extracted cell type.

◊

Reviewer's comment: Figure 4b: What do the box plots show? Could this be described in the caption?

Our reply: In figure 4b, the box plots show the performances for survival outcome prediction for each one of the cohorts (TCGA and IALT). It shows the concordance index when using either only omics data, only histopathology slides or in a multimodal setup. This helps us see the interest of multimodal integration for assessing risk evaluation in Lung Cancer on these particular datasets. Thanks to your comment we updated the caption so that to reference in clearer terms these performance results.

◊

3 Reviewer 2

Reviewer's comment: In line 116, what is the specific clustering algorithm used to divide the patch representations into categories?

Our reply: Thank you for highlighting this, we overlooked to specify the clustering algorithm. In fact, we used a K-Means algorithm for clustering as it was extensively used for WSI region clustering in the literature. It has been clarified.

◊

Reviewer's comment: In line 118, what are Vi and Ei in the defined hypergraph Gi? Xi is defined in line 99, but I don't see definitions for the other two variables.

Our reply: We're sorry that the notations used may be unclear, V_i and E_i are classical notations for standard graph entities, respectively for the vertices and edges of the hypergraph. For the sake of clarity, we defined them properly in the corresponding section.

◊

Reviewer's comment: In lines 128-129, could the authors provide more detail regarding how these gene sets are defined?

Our reply: Here, gene sets are defined from the literature and Multimodal CustOmics takes a list of gene sets as input. In the following analysis, we focused on the Hallmarks of Cancer but the method doesn't lose generality as it can be used with other gene sets as shown in Table S5.

◊

Reviewer's comment: In the "4.1 Prediction Results" section starting on line 285, it would be helpful to note some of the specific performance metrics in the text.

Our reply: Thank you for your suggestion, we added specifications on the metrics in the text as well.

◊

Reviewer's comment: In Figures 2 and 3, the histology slide image on the far left should be labeled/annotated so that the reader can easily identify what is being depicted. The same should be done for the histology images in Figure 4.

Our reply: Thank you for this suggestion to improve figure clarity. We have added annotations to the histology slides in Figures 2, 3, and 4 to clearly label the key morphological features and regions being depicted. This will help readers better understand the visual elements being analyzed in our experiments and their biological significance.

◊

Reviewer's comment: In Figures 2e, 3e, and 4c, the adjusted p-value scale should be labeled with numeric values.

Our reply: Thank you for catching this oversight. We have added numeric values to the adjusted p-value scale in Figures 2e, 3e, and 4c to ensure readers can precisely interpret the statistical significance of the results.

◊

Reviewer's comment: In the supplement, there is nothing listed under the "1 Supplementary Tables" header. Instead, all supplementary text and tables are combined under the "2 Supplementary Texts" header.

Our reply: Thank you for pointing out this mistake, it has been corrected in the revised version of the supplementary materials.

◊

Reviewer's comment: There is some inconsistency in the naming of supplementary tables in the supplement. For example, Tables S5 and S6 are titled "Table 5" and "Table 6."

Our reply: Thank you for highlighting this typo, it has been corrected in the revised version of the supplementary materials.

◊

Reviewer's comment: The supplement includes a "Text S3," but this is not mentioned in the body of the main manuscript or noted under "Supporting information."

Our reply: Thank you for highlighting this mistake, it has been corrected in the revised version.

◊

Reviewer's comment: In the Figure 1 caption, "Multimodal Dropout" is labeled differently from the "Modality Dropout" header in the figure.

Our reply: Thank you, we changed the label in the caption for more consistency in the revised version of the manuscript.

◊

Reviewer's comment: In lines 96 and 149, "inputted" should be changed to "input."

Our reply: Thank you , it has been changed in the revised version.

◊

Reviewer's comment: In Table 1, it would be helpful to bold the CustOmics approaches in the Methods column, like they are in Table 2.

Our reply: Thank you for highlighting this, it has been changed on the revised version of the manuscript

◊

Reviewer's comment: In Figure 2d, there is a halo effect around the numbers at the top and bottom of each plot.

Our reply: Thank you for noticing this subtle detail on the figure, it has been dealt with in the revised version of the manuscript.

◊
